# Blend sign predicts poor outcome in patients with intracerebral hemorrhage

**DOI:** 10.1371/journal.pone.0183082

**Published:** 2017-08-22

**Authors:** Qi Li, Wen-Song Yang, Xing-Chen Wang, Du Cao, Dan Zhu, Fa-Jin Lv, Yang Liu, Liang Yuan, Gang Zhang, Xin Xiong, Rui Li, Yun-Xin Hu, Xin-Yue Qin, Peng Xie

**Affiliations:** 1 Department of Neurology, The First Affiliated Hospital of Chongqing Medical University, Chongqing, China; 2 Department of Neurology, Yongchuan Hospital of Chongqing Medical University, Chongqing, China; 3 Department of Radiology, The First Affiliated Hospital of Chongqing Medical University, Chongqing, China; 4 Department of Radiology, University-Town Hospital of Chongqing Medical University, Chongqing, China; 5 Department of Neurology, Chongqing Jiulongpo People’s Hospital, Chongqing, China; Universitatsklinikum Freiburg, GERMANY

## Abstract

**Introduction:**

Blend sign has been recently described as a novel imaging marker that predicts hematoma expansion. The purpose of our study was to investigate the prognostic value of CT blend sign in patients with ICH.

**Objectives and methods:**

Patients with intracerebral hemorrhage who underwent baseline CT scan within 6 hours were included. The presence of blend sign on admission nonenhanced CT was independently assessed by two readers. The functional outcome was assessed by using the modified Rankin Scale (mRS) at 90 days.

**Results:**

Blend sign was identified in 40 of 238 (16.8%) patients on admission CT scan. The proportion of patients with a poor functional outcome was significantly higher in patients with blend sign than those without blend sign (75.0% versus 47.5%, P = 0.001). The multivariate logistic regression analysis demonstrated that age, intraventricular hemorrhage, admission GCS score, baseline hematoma volume and presence of blend sign on baseline CT independently predict poor functional outcome at 90 days. The CT blend sign independently predicts poor outcome in patients with ICH (odds ratio 3.61, 95% confidence interval [1.47–8.89];p = 0.005).

**Conclusions:**

Early identification of blend sign is useful in prognostic stratification and may serve as a potential therapeutic target for prospective interventional studies.

## Introduction

Intracerebral hemorrhage (ICH) is a common neurological disorder that accounts for approximately 15–30% of all strokes worldwide[1]. It is a major public burden with high morbidity and mortality. The reported 30-day case fatality rate of ICH was around 40%–50%[2–5]. Hematoma volume is the most important prognostic factor in patients with spontaneous ICH[6,7]. Early hematoma expansion has been observed in approximately one third of patients with ICH and is associated with poor functional outcome[8–11]. CT angiography (CTA) spot sign or contrast extravasation has been shown to be a reliable imaging marker that predicts hematoma expansion in patients with ICH[12–15]. However, identification of the CTA spot sign requires iodine contrast administration and early CT angiography examination which is not available in many institutions. It is important to develop imaging predictors for hematoma expansion based on admission CT. Recently, a novel imaging marker termed the CT blend sign has been identified on non-enhanced CT. In a study of 172 patients with ICH, Qi Li et al reported that the blend sign predicts hematoma expansion with high specificity[16]. The prognostic value of CT blend sign has not been fully investigated in patients with ICH. The value of CT blend sign in predicting hematoma expansion has been validated in 784 patients with ICH[17]. Recent studies showed high correlation between CT blend sign and CTA spot sign[18–19]. It remains unknown whether the novel blend sign has independent prognostic significance in patients with ICH. The purpose of our study was to investigate the association of blend sign with the specific outcomes of death and major disability in patients with ICH.

## Methods

### Patient selection

Patients with spontaneous ICH admitted to our hospital between July 2011 and May 2016 were analyzed from our ongoing prospective ICH research database. Patients were eligible for the study if the initial CT scan was performed within 6 hours after the ictus. Patients were excluded from the study if they had secondary ICH due to arteriovenous malformation, rupture of an intracranial aneurysm, traumatic brain injury, brain tumor stroke, or hemorrhagic infarction. Patients were also excluded from the study if they had primary intraventricular hemorrhage. Patients were excluded from the study if they refused to follow-up clinical assessment after discharge from hospital. The demographic data, previous medical history, cigarette smoking, alcohol consumption and medication use were recorded. The admission and in-hospital parameters including Glasgow Coma Scale and blood pressure were assessed. The study was approved by the Ethics Committee of The First Affiliated Hospital of Chongqing Medical University. Written informed consent was obtained from the patients or their legal representatives. The study protocol was conducted in accordance with the declaration of Helsinki.

### Outcome measures

The functional outcome was assessed by using the modified Rankin Scale (mRS) at 90 days. The functional outcome was categorized as favorable and poor outcome. Favorable outcome was defined as mRS of ≤ 2. Patients with mRS ≥ 3 were considered to have poor outcome according to previous studies[20–22].

### Imaging

**Fig 1:**
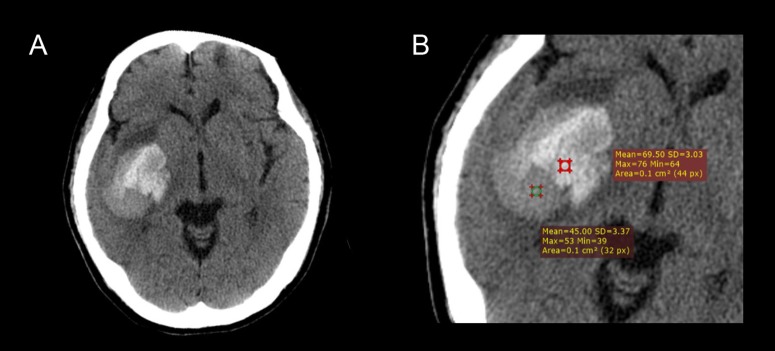
Illustration of CT blend sign. (A) Blend sign appears as a mixed-density hematoma with two well-defined components (a relatively hypoattenuating area and adjacent hyperattenuating region). (B) Further CT densitometry reveals that the hematoma have > 18 Hounsfield unit difference (24.5 HU) between the two density regions.

The diagnosis of ICH was made by admission CT scan with 5mm section thickness. Hematoma growth was defined as an increase in volume >33% or absolute increase of >12.5mL according to previous definitions[16,23–24]. The hematoma volume was calculated by using the ABC/2 formula. Blend sign was defined as hematoma with two well-defined components (a relatively hypoattenuating area and adjacent hyperattenuating region). The hematoma should have > 18 Hounsfield unit difference between the two density regions and the relatively hypoattenuating area was not encapsulated by the hyperattenuating region (Fig 1) [16]. Two readers blinded to the clinical information independently reviewed the CT images. The locations of hematoma were classified as basal ganglia, thalamus, cerebral lobe, brain stem, and cerebellum. Consensus analysis was made by joint discussion in cases of discrepancies.

### Statistical analysis

All statistical analysis was performed with a commercially available software SPSS version 19.0 (SPSS Inc). Data are presented as mean±standard deviations (SD) or as median and interquartile range as appropriate. The frequency distributions of categorized variables were compared between patients with blend sign and those without blend sign by using Fisher exact test, and student’s t test as appropriate. The interobserver agreement was assessed by calculating kappa values. The significance level was set at P<0.05. Stepwise forward inclusion multivariate logistic regression analysis was used to investigate if the blend sign was an independent predictor of mortality and poor functional outcome at 3 months. Variables reaching a statistical trend in univariable analysis (P<0.1) were included in the final model.

## Results

### Prevalence of blend sign and associated factors

A total of 238 patients were included in the final analysis. There were 159 males and 79 females. The average age of the patients was 60.3 years (age range: 27–90). The hematoma volume at presentation was 17.22±15.16 mL. The time from symptom onset to initial CT scan was 2.54±1.73 hours. Hematoma was located in basal ganglia (52.5%), thalamus(26.9%), cerebral lobes(13.0%), brainstem (3.4%) and cerebellum (4.2%).

Hematoma growth occurred in 76 of 238 (31.9%) patients with ICH. Blend sign was observed in 40 of 238 (16.8%) patients on hospital admission CT. Blend sign was located in the basal ganglia (60.0%), thalamus (7.5%), cerebral lobes (30.0%), and cerebellum (2.5%). The inter-observer agreement for identifying blend sign was excellent (κ = 0.91, 95% CI 0.83–0.97).

The baseline demographic, clinical and radiological variables in patient with and those without blend sign were listed in Table 1. The age, gender, history of diabetes, admission blood pressure, smoking and alcohol consumption did not differ significantly between patients with blend sign and those without blend sign (P>0.05).

**Table 1:** Comparison of baseline demographic, clinical, and radiological characteristics between patients with blend sign and those without blend sign.

Variables	Blend Sign Positive (n = 40)	Blend Sign Negative (n = 198)	P Value
**Demographic**			
Mean age, y(SD)	60.0(14.6)	60.4 (11.6)	0.865
Sex, male, n(%)	32(80.0)	127(64.1)	0.052
**Medical history**			
Alcohol consumption, n (%)	22(55.0)	82(41.8)	0.126
Smoking, n (%)	24(60.0)	89(45.4)	0.092
Hypertension, n (%)	25(62.5)	145(73.6)	0.155
Diabetes mellitus, n (%)	3(7.5)	26(13.2)	0.461
Anti-platelet treatment, n (%)	1(2.5)	10(5.1)	0.769
**Clinical features**			
Systolic blood pressure, mmHg (SD)	169.6(28.8)	169.9(28.0)	0.955
Diastolic blood pressure, mmHg (SD)	98.7 (19.9)	97.9(16.5)	0.801
Baseline GCS score, median (IQR)	13(8.25–14)	14(10–15)	0.028
Baseline ICH volume, mL (SD)	28.8 (21.7)	14.9(12.3)	<0.001
Hematoma growth, n (%)	31(77.5)	45(22.7)	<0.001
IVH at baseline CT, n (%)	8(20.0)	69(34.8)	0.067
Speed of bleeding, mL/h, median (IQR)	10.0(4.7–24.4)	6.1(2.8–12.2)	0.002
Hydrocephalus at baseline CT, n (%)	1(2.5)	3(1.5)	0.523
SAH at baseline CT, n (%)	9(22.5)	20(10.1)	0.055
MLS at baseline CT, n (%)	12(30.0)	53(26.8)	0.676
**Outcome**			
In-hospital mortality, n (%)	4(10.0)	9(4.5)	0.316
90-day mRS score, median (IQR)	4(2.25–5)	2(1–5)	0.005
90-day mRS 3–6, n (%)	30(75.0)	94(47.5)	0.001

Abbreviations: SD standard deviation, IQR inter-quartile range, CT computed tomography, GCS Glasgow Coma Scale, ICH intracerebral hemorrhage, IVH intraventricular hemorrhage, mRS modified Rankin Scale, SAH subarachnoid hemorrhage, MLS midline shift.

The sensitivity, specificity, positive predictive value and negative predictive value of blend sign in predicting poor outcome were 24.2%, 91.2%, 75.0%, and 52.5%, respectively.

Patients with blend sign had larger baseline hematoma volume (28.8±21.7 vs 14.9±12.3; P<0.001) and lower GCS score (13(8.25–14) vs 14(10–15); P = 0.028). The speed of bleeding calculated as the initial hematoma volume (mL) divided by time from onset to initial imaging (hours) was significantly faster in patients with blend sign (10.0 mL/h) than those without the sign (6.1 mL/h) (P = 0.002). Radiological parameters such as intraventricular hemorrhage, midline shift, hydrocephalus at presentation and subarachnoid hemorrhage were not statistically different between blend sign positive patients and those without blend sign (*P*>0.05).

### Outcome assessment

**Fig 2:**
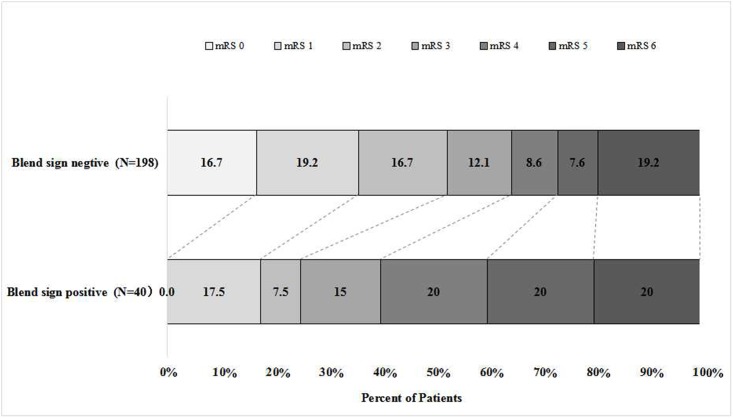
Distribution of modified Rankin Scale at 90 days in patients with blend sign and those without blend sign. The percentage of participants with the modified Rankin scale obtained at 90 days is shown in each cell.

The distribution of modified Rankin Scale at 90 days in patients with blend sign and those without blend sign was illustrated in Fig 2. A total of 124 patients (52.1%) had poor outcome (mRS 3–6) at 3 months. In-hospital mortality is more than doubled (10.0% versus 4.5%; P = 0.316) in blend sign positive patients than those without blend sign. The 90 day mortality was roughly the same between blend sign positive patients and those without blend sign. The proportion of patients with a poor functional outcome was significantly higher in patients with blend sign and those without blend sign (75.0% versus 47.5%, P = 0.001). Interestingly, none of the patients with positive blend sign was free of symptoms at 90 days follow-up.

The results of univariable logistic regression analysis were listed in Table 2. Univariable regression analysis revealed age, intraventricular hemorrhage, baseline hematoma volume, admission GCS score and presence of blend sign on baseline CT scan were associated with poor functional outcome. After controlling for confounding variables, age, intraventricular hemorrhage, admission GCS score and presence of blend sign on baseline CT independently predict poor functional outcome (Table 3).

**Table 2:** Univariable analysis of predictors for poor outcome.

Variable	Odds Ratio	95% Confidence Interval	P Value
Age	1.04	1.02–1.07	<0.001
Current smoking	1.11	0.67–1.86	0.679
Alcohol consumption	0.89	0.53–1.48	0.644
Hypertension	0.90	0.51–1.59	0.723
Systolic blood pressure	1.01	1.00–1.02	0.196
Diastolic blood pressure	1.00	0.99–1.02	0.962
Diabetes mellitus	1.16	0.53–2.54	0.707
Intraventricular hemorrhage	3.48	1.95–6.20	<0.001
Infratentorial hemorrhage	0.91	0.35–2.39	0.913
SAH at baseline CT	1.59	0.72–3.53	0.254
Baseline ICH volume	1.05	1.03–1.08	<0.001
Blend sign on baseline CT	3.32	1.54–7.16	0.002
Antiplatelet use	1.66	0.47–5.83	0.429
Baseline GCS score	0.79	0.72–0.87	<0.001

Abbreviations: ICH intracerebral hemorrhage, GCS Glasgow Coma Scale, CT computed tomography, SAH subarachnoid hemorrhage.

**Table 3:** Multivariate analysis of predictors for poor outcome.

Variable	Odds Ratio	95% Confidence Interval	P Value
Age	1.05	1.02–1.07	0.001
Intraventricular hemorrhage	3.73	1.95–7.13	<0.001
Baseline ICH volume	1.04	1.01–1.07	0.007
Blend sign on baseline CT	3.61	1.47–8.89	0.005
Baseline GCS score	0.86	0.78–0.96	0.004

Abbreviations: ICH intracerebral hemorrhage, GCS Glasgow Coma Scale, CT computed tomography.

## Discussion

This study is the first analysis of the prognostic value of a novel imaging marker termed CT blend sign in patients with ICH. We have demonstrated that CT blend sign is an ominous imaging marker that is associated with poor functional outcome at 3 months.

CT blend sign, which is defined as mixed density hematoma of two well-defined components with > 18 Hounsfield unit difference between the two density regions, was first reported by Qi Li et al in 2015[16]. In a study of 172 patients with ICH, Qi Li and colleagues reported the novel imaging marker predicts hematoma growth. However, the prognostic value of CT blend sign remains unclear.

Li et al proposed that CT blend sign occurs as a result of active bleeding. When clot retracts, the serum was sequestered out of the clot, making the clot hyperintense on admission CT scan[25]. In our study, we have demonstrated that the ultraearly hematoma growth was much faster in patients with blend sign than those without the sign. Our finding may further support Li’s assumption that blend sign may represent clot of different age. The speed of ultra-early hematoma growth was first proposed by Rodriguez-Luna et al and was associated with poor outcome in patients with ICH[26]. In a study of 133 patients with acute (<6 hours) supratentorial ICH, Rodriguez-Luna et al reported that the ultra-early speed of bleeding was significantly faster in spot sign patients as well as in patients who experienced hematoma growth. In a recent pooled analysis of the INTERACT1 and INTERACT2 studies, Sato et al reported a linear association between ultraearly hematoma growth and outcome[27]. More recently, Rodriguez-Luna et al found that the speed of ultraearly hematoma growth was higher in spot sign patients[28]. The association between the presence of CTA spot sign and poor functional outcome has been well established in previous reports[12–15]. In a recent study of 182 patients with spontaneous intracerebral hemorrhage, Sporns et al demonstrated that blend sign is associated with CTA spot sign [18]. In addition, the authors also found that presence of blend sign is an independent predictor of neurological deterioration. However, the association between blend sign and 90 day functional outcome was not assessed in the study. Recently, several non-contrast CT markers of hematoma expansion, such as irregular morphology and hematoma heterogeneity has been proposed [29]. Hematoma sedimentation levels, black hole sign and CT hypodensities have been associated with early hematoma growth [17, 30–31]. More recently, Boulouis et al reported that noncontrast CT hypodensities also predict poor outcome in ICH patients [32]. In our study, we found that the blend sign on admission CT scan predicts poor outcome in patients with ICH. Since CTA spot sign, hematoma sedimentation level and CT blend sign were associated with poor functional outcome, we propose that blend sign, hematoma sedimentation level and CTA spot sign may share common pathophysiological basis-fast ultraearly hematoma growth.

In our study, we have demonstrated that the presence of blend sign on admission CT scan is associated with poor functional outcome as measured by mRS score of 3–6. The difference of poor outcome is mainly attributable to moderately severe disability (mRS = 4) and severe disability (mRS = 5). Notably, none of the patients with positive blend sign was free of symptoms at 90 days follow-up. In this study, we have included patients presented within 6 hours after onset of symptoms. Our previous work suggested that blend sign is associated with hematoma expansion in early ICH patients scanned within 6 hours[16]. The clinical utility of blend sign in predicting hematoma expansion and poor functional outcome remains unclear in ICH patients presented outside 6 hours.

Our study has several limitations. First, patients with very large hematoma and rapid clinical worsening may die before CT scan and would therefore not be included. Second, we used ABC/2 for hematoma volume analysis which is less accurate than modern planimetric techniques. Third, anticoagulant-associated ICH was excluded from our study.

In conclusion, our study demonstrated that blend sign is an ominous imaging marker that is associated with disability at 3 month follow-up in patients with ICH. It is a novel prognostic sign that should be considered in the early evaluation and treatment of patients with ICH.
